# Virtual data augmentation method for reaction prediction

**DOI:** 10.1038/s41598-022-21524-6

**Published:** 2022-10-12

**Authors:** Xinyi Wu, Yun Zhang, Jiahui Yu, Chengyun Zhang, Haoran Qiao, Yejian Wu, Xinqiao Wang, Zhipeng Wu, Hongliang Duan

**Affiliations:** 1 Artificial Intelligence Aided Drug Discovery Institute, College of Pharmaceutical Sciences, Zhejiang University of Technology, Hangzhou, 310014 People’s Republic of China; 2 State Key Laboratory of Drug Research, Shanghai Institute of Materia Medica (SIMM), Chinese Academy of Sciences, Shanghai, 201203 People’s Republic of China; 3 College of Mathematics and Physics, Shanghai University of Electric Power, Shanghai, 201203 People’s Republic of China

**Keywords:** Chemistry, Cheminformatics, Organic chemistry

## Abstract

To improve the performance of data-driven reaction prediction models, we propose an intelligent strategy for predicting reaction products using available data and increasing the sample size using fake data augmentation. In this research, fake data sets were created and augmented with raw data for constructing virtual training models. Fake reaction datasets were created by replacing some functional groups, i.e., in the data analysis strategy, the fake data as compounds with modified functional groups to increase the amount of data for reaction prediction. This approach was tested on five different reactions, and the results show improvements over other relevant techniques with increased model predictivity. Furthermore, we evaluated this method in different models, confirming the generality of virtual data augmentation. In summary, virtual data augmentation can be used as an effective measure to solve the problem of insufficient data and significantly improve the performance of reaction prediction.

## Introduction

Today, organic synthesis occupies a core position in the organic chemistry field and supports the research and development of other fields, such as material science, environmental science, and drug discovery. With increasing advancements in artificial intelligence, several successful applications have been devised in the fields of integrated organic chemistry and artificial intelligence, such as reaction prediction^1–10^. One of the most compelling approaches to predicting reactions is Nam and Kim's proposal to view reaction prediction as a translation task; implemented based on a neural machine translation (NMT) model^5^. Ahneman et al. successfully used machine learning to predict the synthetic reaction performance of Buchwald–Hartwig cross-coupling^7^. Schwaller et al. creatively used sequence-to-sequence models to aid predictions in organic chemistry^10^.

Deep learning, a popular branch of artificial intelligence has considerably progressed in areas such as speech recognition, visual object recognition, and other fields such as organic reaction prediction^11–13^. However, deep learning methods are generally determined using large datasets. In addition, previous research has demonstrated that focusing on massive reaction datasets requires considerable effort^14^. Moreover, in all these studies, the amount of data considered for a particular reaction type was insufficient to support related applications because of high costs and time-consuming experiments. Therefore, deep learning methods must be able to comprehensively deal with small datasets to solve project-tailored tasks in the cross-domain with chemistry.

As such, many strategies have been designed for improving the poor performance in the small datasets of deep learning methods^15–20^. An effective method is transfer learning, which transfers prior knowledge, learned from abundant data, to another domain task; this can subsequently be used in situations with less data but similar task scenarios^21–23^. Reymond et al. performed transfer learning on carbohydrate reactions and showed better performance than a model trained only on carbohydrate reactions^23^. Apart from the transfer learning method, data augmentation strategies are crucial for deep learning pipelines aiming at reaction prediction tasks, as the performance of the model increases with the amount of training data. Data augmentation is the process of modifying or “augmenting” a dataset with additional data; this is a powerful strategy used in image processing^24–26^. Tetko et al. proved that augmenting input and target data simultaneously can improve the performance of predicting new sequences^27^. In general, augmenting training-set sequences allows deep learning methods to achieve better accuracy according to the characteristic of the simplified molecular-input line-entry system (SMILES)^28,29^. Notably, all the augmented SMILESs are valid structures without changing their chemical meaning. Inspired by the work of Maimaiti et al., we propose an intelligent strategy for data augmentation; they manually created a batch of fake data to increase the target training set by deleting words, randomly sampling words, or replacing some words during text generation^30^. In this manner, synthetic data augmentation was realized by transforming the text into low-resource language scenarios. Based on the similarity of the SMILES representation to the text, we added fake data instead of “random SMILES” to the training dataset to improve model accuracy, terming it as virtual data augmentation. The fake data was generated by replacing substituents with equivalent functional groups in the reactants, which do not change the reaction sites and atom valences of the reactant molecules.

**Figure 1:**
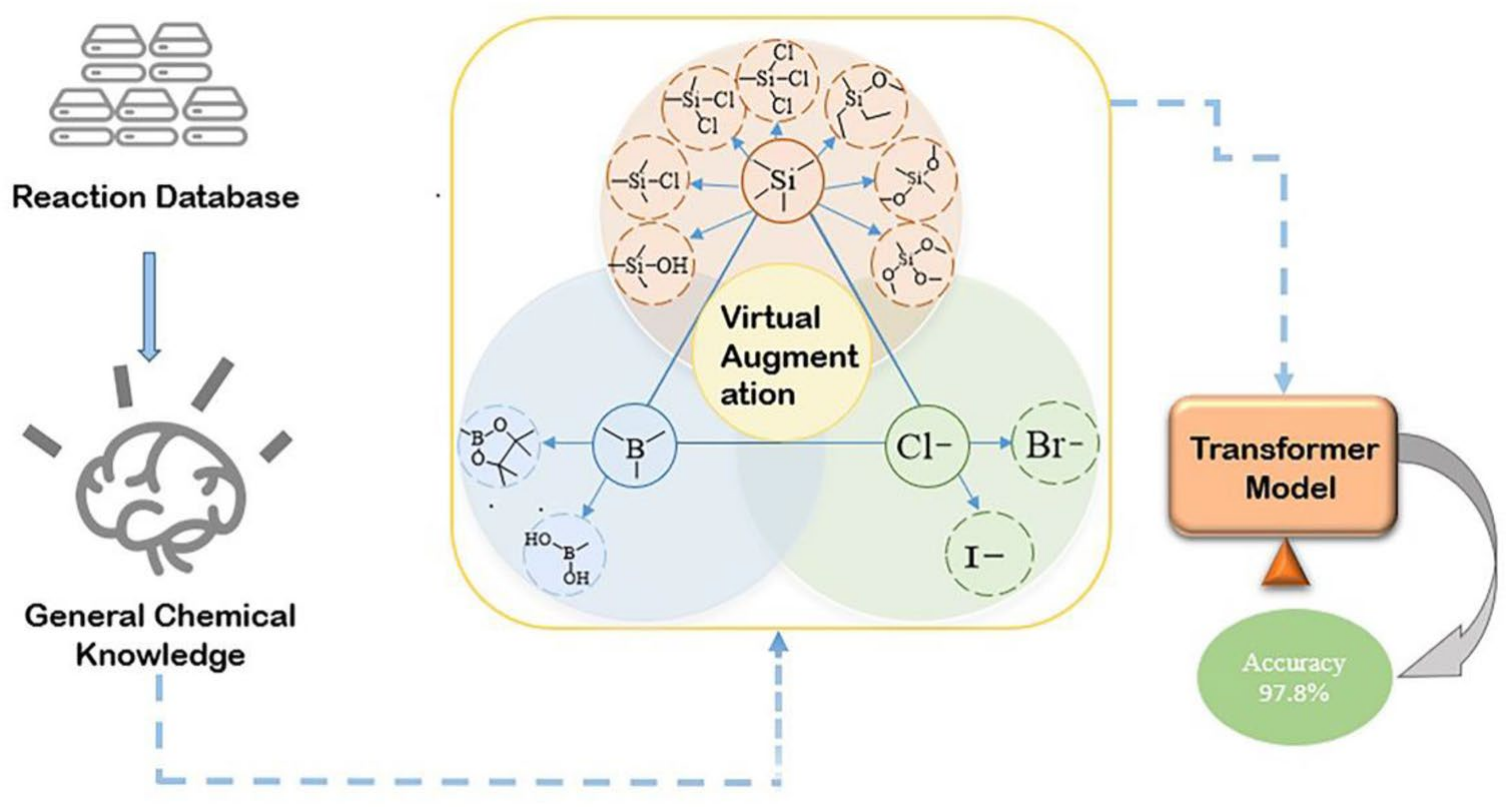
Schematic illustration of the virtual data augmentation method.

In this study, we applied and scrutinized virtual data augmentation. In addition, we show that the fake data can lead to better performance on the transformer model, which is state-of-the-art in natural language processing^27,31,32^. Although the transformer model shows excellent performance in various reaction tasks, the data-driven model remains inefficient in the case of insufficient data resources. Our study was aimed at predicting the outcomes of reactions, as detailed in Fig. 1. The datasets used in this study are coupling reactions; an organic chemical reaction in which two chemical entities (or units) combine to form one molecule. When the virtual data augmentation method is trained on the transformer-baseline model, the accuracy of reaction prediction compared with the raw data is improved from 2.74 to 25.8%. Furthermore, combined with the transfer learning method, the performance of the transformer model increased from 1 to 53%, proving that this virtual data augmentation can improve the model performance. Overall, this virtual data augmentation aims to expand the density of sample data points in the chemical space already covered by the existing literature datasets. Moreover, we believe that this method can be a useful tool for solving tasks with small datasets using deep learning methods in low-resource scenarios.

## Methods

### Dataset preparation

In this study, we exported five coupling reaction datasets, i.e., those of Buchwald–Hartwig, Chan–Lam, Kumada, Hiyama, and Suzuki’s, based on the name and structure search from the ‘Reaxys’ database^33^. Each dataset was preprocessed as follows. First, irrelevant information (e.g., pressure, temperature, yield, etc.) was omitted from these datasets, retaining only reaction and reagent entries. Secondly, the reaction SMILES were canonized, and all the duplicated reaction entries were removed. Finally, the five reaction datasets were filtered using template screening based on the respective reaction rule (Supplementary Fig. S1).

**Figure 2:**
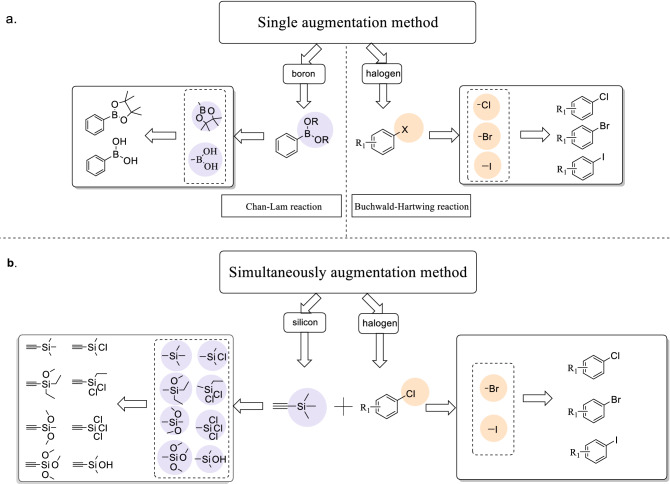
The schematic diagram of virtual data augmentation. (**a**) The single augmentation method of Buchwald-Hartwig and Chan-Lam coupling reactions. (**b**) The representative example of simultaneously virtual data augmentation method of Hiyama coupling reaction.

Next, the virtual data augmentation method was divided into two types according to the general characteristics of reactants in the five coupling reactions. The fake dataset was created by replacing some functional groups of the same kind, for example, if the reactants contain the functional group chlorine, our strategy was to replace it with bromine or iodine, which are also halogen functional groups. That is, the fake data are compounds with modified functional groups. Therefore, the first augmentation method replaces the functional group of one of the reactants, defined as a single augmentation in this study. As shown in Fig. 2a, for the Chan–Lam reaction, the virtual data augmentation was carried out in the reactants with the boron functional group, whereas the Buchwald–Hartwig reaction augmented the reactants with the halogen functional group. The other virtual data augmentation method involves the simultaneous substitution of functional groups for multiple reactants; this is termed simultaneous augmentation. By considering the Hiyama as an example (Fig. 2b), the reaction augments the reactants with the silicon and halogen functional groups simultaneously. Further, the Kumada reaction simultaneously augmented the reactants and Grignard reagents with the halogen functional groups, and the Suzuki reaction augmented the reactants with halogen and boron functional groups simultaneously.

In addition, Table 1 lists the statistical summary of the five coupling reactions before and after data augmentation and describes the corresponding reaction formulas. The virtual data augmentation method significantly increased the amount of data by approximately two to six times that of the raw data.

**Table 1:** Statistical Summary of five coupling reactions before and after using virtual data augmentation method.

Name	Depiction	Raw dataset	Virtual dataset
Hiyama		2067	19011
Buchwald-Hartwig		4419	7640
Chan-Lam		5276	9170
Kumada		9657	54062
Suzuki		92399	424194

Ultimately, for the augmented dataset and raw dataset, we randomly divided the datasets into training, validation, and test datasets at a ratio of 8:1:1. To avoid contingency, we augmented the training dataset of these five reactions without augmenting validation and test datasets. It is worth noting that the repeated reaction produced by the virtual data augmentation method has been deleted. In addition, all scripts are written in Python (version 3.7) and using RDKit for processing^34^.

### The U.S. Patent and Trademark Office (USPTO) dataset

The data we used to pretrain the model were derived from Lowe's patent mining work, in which the reactions were extracted from the USPTO patented reactions granted between 1976 and 2016, as available to the public^35^. Next, Coley et al. extracted 480k reactions from the USPTO-authorized patents^36^. We processed these data, deleted reaction reagents and chirality, and filtered out incomplete or wrong reactions. Furthermore, after pretreatments, such as standardization and repeat removal, we recovered chirality from the USPTO for each reaction originally containing chirality. Note that we extracted only one product of the reaction, and the raw and augmented datasets were removed from the USPTO dataset. Finally, about 410k single-product reactions were obtained as a pretraining dataset.

### Model

During the work, the transformer baseline and transformer transfer learning were mainly adopted to verify the validity of the augmentation method.

The proposed model is based entirely on the transformer model, which is a powerful model for handling natural language processing (NLP) tasks and was devised by Google in 2017^20^. The reaction prediction model by Zhang et al. was selected as the baseline model in our work^37^. This model solely relies on the attention mechanism to handle text tasks without the use of recurrent neural networks (RNNs) and convolutions, and it does not encounter the recursion problem that exists in encoder–decoder architectures. Moreover, the model contains several identical encoder–decoder layers. In addition, the application of multiheaded attention (MHA) in the decoder increases the computation speed and improves model performance. The reaction prediction process of the transformer model is as follows: (1) the reactants are transmitted to the encoder in the form of SMILE codes as input; (2) these codes are then transferred to the next encoder until the last encoder transmits them to the decoder; (3) finally, the decoder outputs the predicted results.

Furthermore, we selected two published reaction prediction models to verify the generality of this augmentation method. The first is the molecular transformer model proposed by Philips et al.^38^. Compared with the seq-2-seq model used for reaction prediction, the RNN component of the molecular prediction model is completely removed and the model itself is based entirely on an attention mechanism, comprising a combination of an MHA layer and a position feedforward layer. The second is the RNN model by Liu et al., which is a fully data-driven model. This model is trained end-to-end and has an encoder–decoder architecture comprising two RNNs^2^.

We also introduced the transfer learning strategy into the transformer model. During the pretraining process, a large chemical reaction dataset, USPTO-410k, was used to pretrain the model. The model transfers the general chemistry information learned from pretraining to the target task of predicting the outcomes of the five coupling reactions. In addition, transfer learning combined with virtual data augmentation further improves the model’s performance. With this new strategy, the model can abundantly learn chemical information from the USPTO-410k dataset and fake data added to the training dataset.

The following hyperparameters were used by the baseline model for reaction predictions:

- optimize adam:
- beta1 = 0.9, beta2 = 0.997
- epsilon = 1e−9
- n_heads = 8
- emb_dim = 256
- num_layers = 6.

## Result

In this study, the transformer model was used to predict the outcomes of several coupling reactions. As the virtual data augmentation method has been efficiently used in transformer models, it is desirable to visualize the relation location of datasets in the chemical space and can determine how the model allows interpretation. We first visualized raw and augmented data among the five reactions by using uniform manifold approximation and projection (UMAP) and the tree-map (TMAP)^39,40^. In addition, the program for drawing the UMAP is publicly available on GitHub, and the map was drawn using the TMAP open-source software^41^.

**Figure 3:**
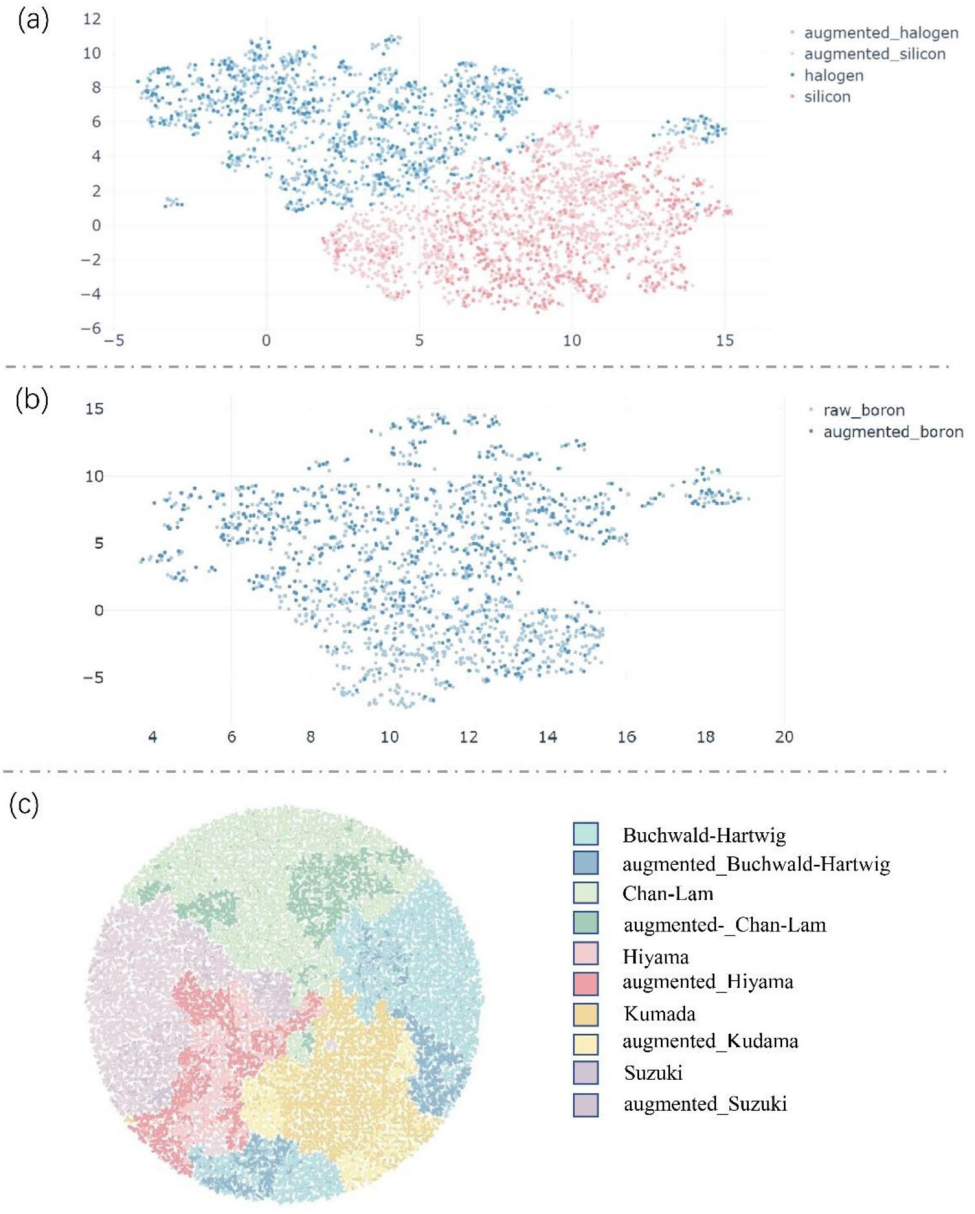
UMAP plot of molecules from raw data and virtual augmented data and TMAP plot of rxnfp of reactions from raw data and virtual augmented data. (**a**) UMAP map of Hiyama coupling reaction before and after virtual data augmentation. (**b**) UMAP map before and after virtual data augmentation of Chan-Lam coupling reaction. (**c**) TMAP before and after virtual data augmentation of five classic coupling reaction.

This section reports on how we generated the plots of reactant molecules of raw and augmented datasets using UMAP, which represents molecules as Morgan fingerprints to create a two-dimensional representation of high-dimensional data distributions. In the following text, we explicate our work by taking the Hiyama and Chan–Lam reactions as examples. As shown in Fig. 3a, the silicon- and halogen-containing molecules generated by virtual data augmentation in the training set of the Hiyama reactions (i.e., light pink and light blue, respectively) are close to the Hiyama raw datasets (pink and blue), respectively. In addition, as shown in Fig. 3b, we generated the UMAP plot of Chan–Lam reactions, which displays only boron-containing molecules that can be augmented individually. The boron-containing molecules generated by virtual data augmentation (blue) are still near the raw datasets (light blue). The graphical analysis presented in the supplementary data (Figs. S2–S4) confirms the effectiveness of virtual data augmentation based on text replacement for maneuvering in a chemical space from the source to the objective.

Additionally, to further explore the relationship of the datasets we used, all the datasets except USPTO-410k were visualized by TMAP. TMAP is another powerful visualization tool to represent large high-diversional datasets as a two-diversional connected tree. In this TMAP plot, both raw and augmented reactions represent a point according to the reaction fingerprint, RXNFP, which is derived from a neural network trained to classify patent chemical reactions. Notably, the data we inserted into TMAP comprised 5000 reactions randomly selected from the data of five coupling reactions before and after amplification. If the raw dataset comprised less than 5000 reactions, we placed all the data into the visualization tool. As shown in Fig. 3c, the raw and augmented datasets derived from the same type of reactions overlapped considerably, and reactions of different types could be separated, illustrating that the fake data produced by the proposed virtual data augmentation method is relatively similar to the raw data. Overall, from the perspective of the training model, the addition of the fake data derived from virtual data augmentation according to text replacement is effective and creates a positive effort in improving the performance of deep learning models.

To avoid the occurrence of overfitting or underfitting, the data were split using 10-fold cross-validation on the transformer-baseline model. The detailed accuracies of each experiment can be found in Supplementary Tables S1–S5, the virtual data augmentation method was first tested on the Hiyama coupling dataset, the smallest dataset of the five reaction datasets. As shown in Table 2, show the average accuracy of Hiyama, Buchwald–Hartwig, Chan–Lam, Kumada, and Suzuki reactions based on the transformer-baseline model. The model was trained with the raw dataset as the training set and we obtained accuracies of 24.96%, 30.09%, 60.99%, 78.52%, and 94.33%, respectively. The use of the transformer model to predict the test set with the augmented training set resulted in higher top-1 predictions of 46.56%, 46.82%, 66.07%, 83.66, and 96.48%, respectively. Overall, although the proposed model achieved an unfavorable performance in predicting outcomes of these five reactions, a distinct increment was observed before and after the training dataset was augmented. We observed that the addition of fake data to expand the datasets can assist the model to improve its predictive performance.

**Table 2:** Average accuracy comparison of several coupling reactions between raw data and augmented data based on the transformer-baseline model.

Dataset	Average accuracy (%)				
	Hiyama	Buchwald–Hartwig	Chan–Lam	Kumada	Suzuki
Raw data	24.96	30.09	60.99	78.52	94.33
Augmented data	46.56	46.82	66.07	83.66	96.48

To verify whether this virtual data augmentation is effective, the transfer learning method was integrated with the transformer-baseline model. In Table 3, we chose k = 1 to future analyze the difference between the transformer-baseline model and transformer-transfer model. Table 3 summarizes the accuracy of these five reactions in the transformer-transfer learning model. On the one hand, with the introduction of the transfer learning strategy, the overall accuracy of the transformer-transfer learning model was improved by nearly 20% on average compared with the transformer-baseline model. On the other hand, the accuracy gaps between the raw and augmented datasets showed considerable improvement with the combination of the transfer learning method, especially in the Buchwald–Hartwig dataset. These results indicate that the combination of fake-data addition and transfer learning can solve the problem of data scarcity. Moreover, the addition of fake data to the training dataset shows superior performance compared with those of raw datasets, indicating that this virtual data augmentation method is effective and can be generalized.

**Table 3:** Accuracy comparison of several coupling reactions between raw data and augmented data based on the transformer-baseline model and transformer-transfer model.

Model	Dataset	Reaction types				
		Hiyama	Buchwald–Hartwig	Chan–Lam	Kumada	Suzuki
Transformer-baseline model	Raw data	23.67	41.63	64.71	78.99	95.05
	Augmented data	49.47	49.32	68.50	85.40	97.79
Transformer-transfer model	Raw data	60.87	94.57	96.39	96.48	97.84
	Augmented data	69.57	95.93	96.77	97.00	98.63

Next, we used the Chan–Lam reaction as an example to show a comparison with previously studied direct generative models. The Molecular Transformer model by Philippe et al. and the neural sequence-to-sequence model by Liu et al. were employed to evaluate the applicability of our virtual data augmentation method^2,38^. As shown in Table 4, the overall performance of the transformer model is significantly higher than that of the RNN model. The accuracy of the two transformer models is nearly 10% higher than that of the RNN model, and the prediction performance of our proposed model does not differ much from that of Philippe et al*.*

**Table 4:** The reaction prediction accuracy of Chan-Lam reaction before and after augmentation was compared under different models.

Dataset	Accuracy (%)		
	RNN	Molecular transformer	Baseline transformer
Raw data	54.08	68.88	64.71
Augmented data	59.38	71.92	68.50

## Discussion

**Figure 4:**
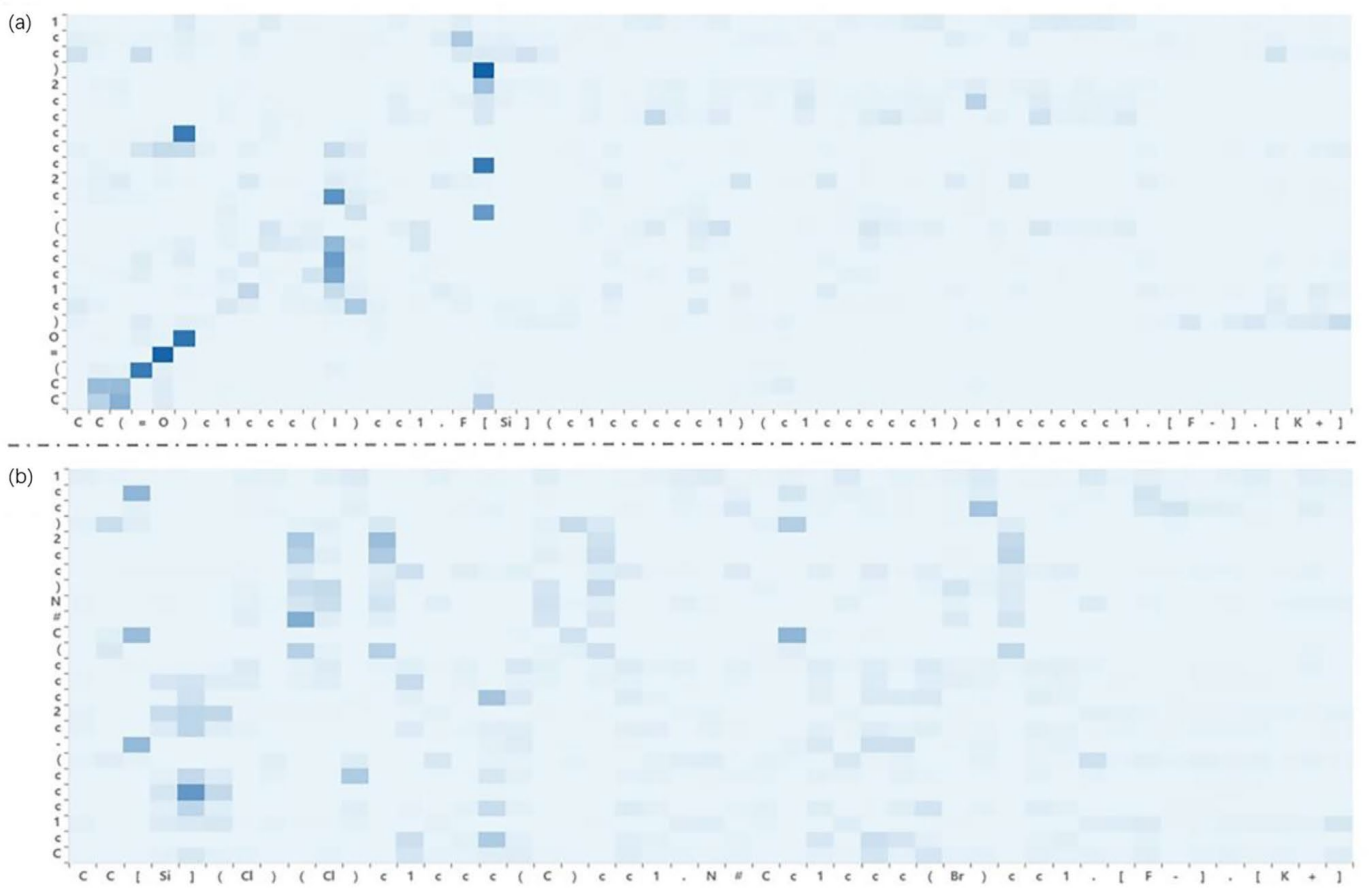
Visualization of attention weight before and after Hiyama reaction augmentation. The horizontal axis contains two reactants and reagents, and the vertical axis is the product. (**a**) SMILES:CC(=O)c1ccc(I)cc1.F[Si](c1ccccc1)(c1ccccc1)c1ccccc1.[F].[K+]>>CC(=O)c1ccc(c2ccccc2)cc1. (**b**) SMILES:CC[Si](Cl)(Cl)c1ccc(C)cc1.N#Cc1ccc(Br)cc1.[F-].[K+]>>Cc1ccc(-c2ccc(C#N)cc2)cc1.

We used attention weight to visualize the learning process of the transformer model^42^. Attention weight is the key to accounting for long-distance dependencies and has been used in reaction predictions and other fields. To predict the outcomes of variable coupling reactions, specific reagents have a certain impact on the model output. Particularly, the use of attention weights can provide a straightforward explanation of how the model learns the inputs and outputs of SMILES. Figure 4a shows the visualization of the attention weight of a set of raw Hiyama reactions. The darker the token, the more noticeable it is in this layer or output step. As shown in Fig. 4, [F–] in the reagent activates the Si–R bond with low polarization in silicone, which exchanges with the R–X, resulting in a cross-coupling reaction. Figure 4b shows a set of reactions in Hiyama augmentation, where the weight of attention in a reaction is almost the same, mainly focusing on the locations where cross-coupling occurs. These results show that there was no difference between the reaction sites of the augmented Hiyama data and the raw data. This implies that our proposed fake data is meaningful for model training.

The virtual data augmentation method is divided into two types. In addition to augmenting both reactants in simultaneous augmentation (Table 1), we performed augmentation experiments based on the reactants in the reactions. For these reactions, we replaced the functional groups of one reactant, and then replaced the other. The results are shown in Table 5. For Hiyama reactions, the reactants were augmented with halogen, and the transformer-baseline model achieved 44.44% accuracy, and for reactants augmented with silicon, the accuracy is comparable with the former. While we performed the augmentations simultaneously, the performance of the transformer-baseline model increased by nearly 5%. A similar phenomenon was observed in the Kumada and Suzuki reactions. Specifically, for the Kumada reaction, the performance of the transformer-baseline model increased from 80.85 to 85.40% after simultaneous augmentation of the reactants. In the case of the Suzuki reaction, it is the largest amount of data in the five response datasets; therefore, the accuracy rate did not improve considerably. However, the overall accuracy rate still improved to nearly 98%. After augmenting all the reactants, the transformer model can learn more chemical information about the reaction, thus achieving higher performance in reaction prediction. This can be attributed to the transformer model’s ability to encode and decode text sequences. Moreover, to understand model training, we also conducted several experiments in which the different number of training datasets were randomly selected to monitor their predictive performance in the transformer-baseline model (Supplementary Table S6).

**Table 5:** The comparisons of different augmented reactants.

Reaction types	Accuracy (%)		
	Augmented halogen	Augmented silicon (or boron)	Simultaneously augmented
Hiyama	44.44	48.31	49.47
Kumada	80.85	84.68	85.40
Suzuki	96.82	95.26	97.79

**Figure 5:**
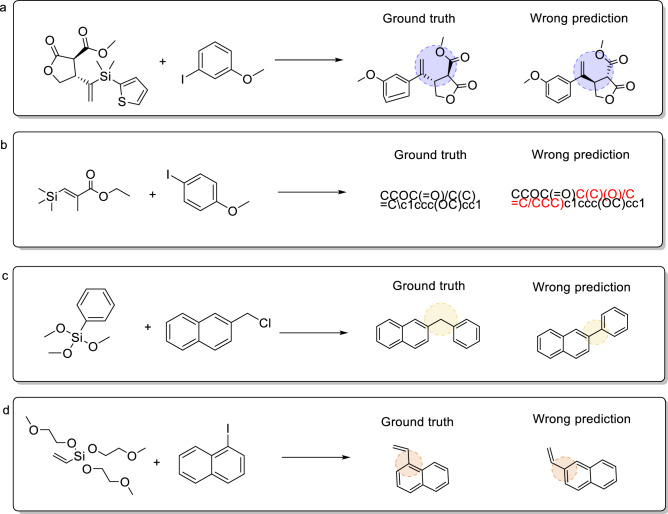
Typical error analysis of Hiyama coupling reactions. (**a**) chirality errors, (**b**) SMILES errors, (**c**) the number of atom errors (**d**) functional group isomerism errors.

**Figure 6:**
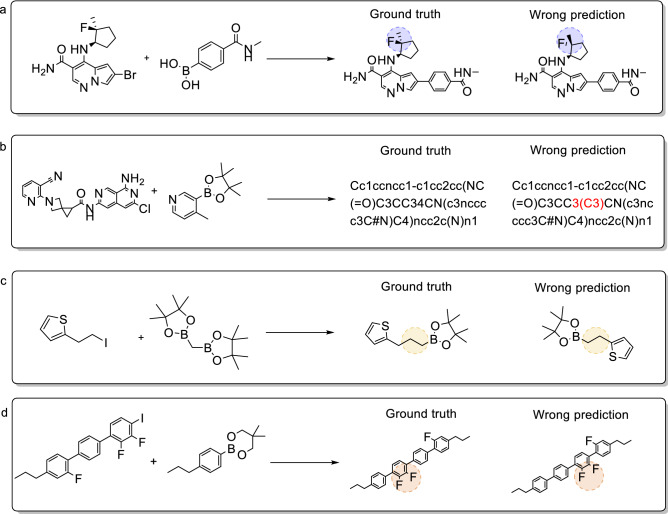
Typical error analysis of Suzuki coupling reactions. (**a**) Chirality errors, (**b**) SMILES errors, (**c**) the number of atom errors, (**d**) functional group isomerism errors.

We further analyzed the incorrect predictions predicted by the transformer-baseline model for these five reactions before and after adding fake data to the training set to evaluate the validity of virtual data augmentation. Four main errors are listed in Table 6: invalid SMILES errors, the number of atom errors, chirality errors, and functional group isomerization errors. Figures 5 and 6 respectively list several representative examples of the Hiyama and Suzuki reactions. Among these four errors, regardless of whether the training was conducted on the raw or augmented datasets, the most common error besides other prediction errors was the SMILES error. The functional group isomerization error is ranked second, particularly for the Hiyama reaction. This is attributable to the fact that our proposed data augmentation method is based on the replacement variable functional group, which causes the functional groups to misunderstand the predictive performance of the model. The other two errors were observed in multiple reaction pre-projects. These errors result in the transformer-baseline model putting forward a modest performance in tackling small reactions. Furthermore, when comparing the amounts of wrong predictions of raw data to the errors of the augmented dataset, we found that the use of virtual data augmentation reduced the ratio of each error by nearly 20%. This indicates that our proposed method improved the model performance from the source.

**Table 6:** The number of error types in reaction prediction for five coupling reactions.

Wrong type	Hiyama lift rate (%)	Suzuki lift rate (%)	Buchwald–Hartwig lift rate (%)	Cham–Lam lift rate (%)	Kumada lift rate (%)
Chirality error	1.00	5.50	1.63	1.71	4.05
SMILES error	11.65	33.50	33.06	28.57	28.38
Group isomerism error	10.67	9.50	15.51	10.86	13.51
Number of carbon error	16.50	11.00	11.43	10.29	16.22
Other’s error	60.19	40.50	38.37	48.57	37.84

## Conclusion

This study reports that our innovative data augmentation method can improve the performance of a transformer model by augmenting the data size of the training set. The model was trained to learn more latent chemical information in organometallic coupling reactions by equivalently replacing groups in reactants corresponding to the raw datasets. This allowed the expansion of the training set and increased the predictive performance of the model. This concept has been intensively used in the field of image recognition; however, it is yet to be used in solving chemical problems^43,44^.

This is the first study to report on the application of virtual data augmentation in the chemical reaction field. We determined that the addition of fake data at the chemical level boosts the predictive performance in reaction prediction. In addition, we verified that our proposed method can be generalized to other models. Moreover, we determined that the virtual data augmentation method combined with the transfer learning strategy can achieve better prediction accuracy. Additionally, we used visualization tools to represent the effectiveness of the virtual data augmentation method and applied attention weight to visualize the prediction process. The accurate visualization demonstrated that virtual data augmentation is meaningful at the chemical level and that this model becomes more sensitive to the selection of reaction sites. In summary, this study demonstrates that the transformer model is suitable for small-scale reactions, and this work opens new possibilities for data augmentation methods. Moreover, this study provides an important milestone in improving the reaction prediction performance on small datasets. Owing to the lack of available institutional data, the development of integrating deep learning methods with the chemical field may be limited. However, the above-mentioned results all confirm that the proposed virtual data augmentation strategy can contribute to reaction prediction based on small datasets. We believe that this method can be applied to other tasks with limited datasets by augmenting the training dataset.

## Supplementary Information

Supplementary Information.

## Data Availability

The dataset (pretraining and self-built) presented in this paper are publicly available on GitHub at https://github.com/hongliangduan/Virtual-data-augmentation-methood-for-reaction-prediction-in-small-dataset-scenario.

## Code availability

All scripts used to process the data^33^ are publicly available on GitHub at https://github.com/hongliangduan/Virtual-data-augmentation-methood-for-reaction-prediction-in-small-dataset-scenario. Python 3.7 version to write the program. RDKit 2020.09.5 version to process programs^34^. The map was drawn using the TMAP open-source software^41^.

## References

[1] Segler M, Preuss M, Waller M (2018). Planning chemical syntheses with deep neural networks and symbolic AI. Nature.

[2] Liu B, Ramsundar B, Kawthekar P (2017). Retrosynthetic reaction prediction using neural sequence-to-sequence models. ACS Cent. Sci..

[3] Baylon JL, Cilfone NA, Gulcher JR (2019). Enhancing retrosynthetic reaction prediction with deep learning using multiscale reaction classification. J. Chem. Inf. Model..

[4] Coley CW, Thomas DA, Lummiss JAM (2019). A robotic platform for flow synthesis of organic compounds informed by AI planning. Science.

[5] Nam, J. & Kim, J. Linking the neural machine translation and the prediction of organic chemistry reactions. Preprint at https://arxiv.org/abs/1612.09529 (2016).

[6] Coley CW, Barzilay R, Jaakkola TS, Green W, Jensen KF (2017). Prediction of organic reaction outcomes using machine learning. ACS Cent. Sci..

[7] Ahneman DT, Estrada JG, Lin S, Dreher SD, Doyle AG (2018). Predicting reaction performance in C-N cross-coupling using machine learning. Science.

[8] Schwaller, P., Laino, T., Gaudin, T., Bolgar, P., Bekas, C. & Lee, A. Molecular transformer for chemical reaction prediction and uncertainty estimation. Preprint at 10.26434/chemrxiv.7297379.v2 (2019). 10.1021/acscentsci.9b00576 PMC6764164 31572784

[9] Baum ZJ, Yu X, Ayala PY, Zhao Y, Watkins SP, Zhou QQ (2021). Artificial intelligence in chemistry: Current trends and future directions. J. Chem. Inf. Model..

[10] Schwaller P, Gaudin T, Lanyi D, Bekas C, Laino T (2018). “Found in Translation”: Predicting outcomes of complex organic chemistry reactions using neural sequence-to-sequence models. Chem. Sci..

[11] Deng, L., Hinton, G. & Kingsbury, B. New types of deep neural network learning for speech recognition and related applications: An overview. in *2013 IEEE International Conference on Acoustics, Speech and Signal Processing* 8599–8603. 10.1109/ICASSP.2013.6639344 (2013).

[12] Cichy RM, Khosla A, Pantazis D, Torralba A, Oliva A (2016). Comparison of deep neural networks to spatio-temporal cortical dynamics of human visual object recognition reveals hierarchical correspondence. Sci. Rep..

[13] Fooshee D (2018). Deep learning for chemical reaction prediction. Mol. Syst. Des. Eng..

[14] Thakkar A, Kogej T, Reymond JL (2020). Datasets and their influence on the development of computer assisted synthesis planning tools in the pharmaceutical domain. Chem. Sci..

[15] Fortunato ME, Coley CW, Barnes BC (2020). Data augmentation and pretraining for template-based retrosynthetic prediction in computer-aided synthesis planning. J. Chem. Inf. Model..

[16] Dao, T., Gu, A., Ratner, A., Smith, V., Sa, C. D. & Ré, C. A kernel theory of modern data augmentation. Preprint at 10.48550/arXiv.1803.06084 (2019). PMC6879382 31777848

[17] Lee AA, Yang Q, Sresht V (2019). Molecular transformer unifies reaction prediction and retrosynthesis across pharma chemical space. Chem. Commun..

[18] Moret M, Friedrich L, Grisoni F (2020). Generative molecular design in low data regimes. Nat. Mach. Intell..

[19] Schwaller, P., Vaucher, A. C., Laino, T. & Reymond, J. L. Data augmentation strategies to improve reaction yield predictions and estimate uncertainty. Preprint at 10.26434/chemrxiv.13286741.v1 (2020).

[20] Tetko IV, Karpov P, Bruno E, Kimber TB, Godin G (2019). Augmentation is what you need!. ICANN.

[21] Smith JS, Nebgen BT, Zubatyuk R (2019). Approaching coupled cluster accuracy with a general-purpose neural network potential through transfer learning. Nat. Commun..

[22] Cai CJ, Wang SW, Xu YJ (2020). Transfer learning for drug discovery. J. Med. Chem..

[23] Pesciullesi G, Schwaller P, Laino T, Reymond JL (2020). Transfer learning enables the molecular transformer to predict regio-and stereoselective reactions on carbohydrates. Nat. Commun..

[24] Simard PY, Steinkraus D, Platt JC (2003). Best practices for convolutional neural networks applied to visual document analysis. ICDAR.

[25] Mikołajczyk, A. & Grochowski, M. Data augmentation for improving deep learning in image classification problem. in *IIPhDW-2018* 117–122. 10.1109/IIPHDW.2018.8388338 (2018).

[26] Alexey D, Fischer P, Tobias J, Springenberg MR, Brox T (2016). Discriminative unsupervised feature learning with exemplar convolutional neural networks. IEEE Trans. Pattern Anal. Mach. Intell..

[27] Tetko IV, Karpov P, Van Deursen R, Godin G (2020). State-of-the-art augmented NLP transformer models for direct and single-step retrosynthesis. Nat. Commun..

[28] Weininger D (1988). SMILES, a chemical language and information system. 1. Introduction to methodology and encoding rules. J. Chem. Inf. Comp. Sci..

[29] Weininger D, Weininger A, Weininger JL (1989). SMILES. 2. Algorithm for generation of unique SMILES notation. J. Chem. Inf. Comp. Sci..

[30] Maimaiti M, Liu Y, Luan H, Pan Z, Sun M (2021). Improving data augmentation for low-resource NMT guided by POS-tagging and paraphrase embedding. ACM Trans. Asian Low-Resour. Lang. Inf. Process..

[31] Xie, Z., Wang, S. I., Li, J., Lévy, D., Nie, A., Jurafsky, D. & Andrew Y, N. Data noising as smoothing in neural network language models. Preprint at 10.48550/arXiv.1703.02573 (2017).

[32] Zheng SJ, Rao JH, Zhang ZY, Xu J, Yang YD (2020). Predicting retrosynthetic reactions using self-corrected transformer neural networks. J. Chem. Inf. Model..

[33] http://www.elsevier.com/online-tools/reaxys.

[34] http://www.rdkit.org.

[35] Lowe DM (2012). Extraction of Chemical Structures and Reactions from the Literature.

[36] Jin, W., Coley, C. W., Barzilay, R. & Jaakkola, T. Predicting organic reaction outcomes with weisfeiler-lehman network. Preprint at https://hdl.handle.net/1721.1/130478 (2017).

[37] Zhang, C. Y., Cai, X. & Qiao, H. R. *et al.* Self-supervised molecular pretraining strategy for reaction prediction in low-resource scenarios. Preprint at 10.26434/chemrxiv-2021-fxvwg (2021).

[38] Schwaller P, Laino T, Gaudin T (2019). Molecular transformer: A model for uncertainty-calibrated chemical reaction prediction. ACS Cent. Sci.

[39] McInnes, L., Healy, J. & Melville, J. Umap: Uniform manifold approximation and projection for dimension reduction. Preprint at 10.48550/arXiv.1802.03426 (2018).

[40] Becht E, McInnes L, Healy J (2019). Dimensionality reduction for visualizing single-cell data using UMAP. Nat. Biotechnol..

[41] https://tmap.gdb.tools/

[42] Schwaller P, Probst D, Vaucher AC (2021). Mapping the space of chemical reactions using attention-based neural networks. Nat. Mach. Intell..

[43] Cireşan DC, Meier U, Gambardella LM, Schmidhuber J (2010). Deep, big, simple neural nets for handwritten digit recognition. Neural Comput..

[44] Dosovitskiy A, Springenberg JT, Riedmiller M, Thomas B (2014). Discriminative unsupervised feature learning with convolutional neural networks. IEEE Trans. Pattern Anal..

