# Supplementary material for: Virtual data augmentation method for reaction prediction
Source: Sci Rep. 2022 Oct 12;12:17098. doi: 10.1038/s41598-022-21524-6 (PMC9556613; doi:10.1038/s41598-022-21524-6)
Supplement: Supplementary file 1 — Supplementary Information. [file 41598_2022_21524_MOESM1_ESM.docx]

**Electronic Supplementary Material (ESI) for Scientific Reports.**

**This journal is © Nature 2022**

**Supporting Information**

Virtual data augmentation method for reaction prediction

Xinyi Wu, ‡^a^ Yun Zhang, ‡^a^ Jiahui Yu, ^a^ Chengyun Zhang, ^a^ Haoran Qiao,^c^ Yejian Wu, ^a^ Xinqiao Wang,*^a^ Zhipeng Wu ^a^ and Hongliang Duan*^a,b^

^a^Artificial Intelligence Aided Drug Discovery Institute, College of Pharmaceutical Sciences, Zhejiang University of Technology, Hangzhou, 310014 (P. R. China).

^b^State Key Laboratory of Drug Research, Shanghai Institute of Materia Medica (SIMM), Chinese Academy of Sciences, Shanghai 201203, China.

*Email: <hduan@zjut.edu.cn>

^c^College of Mathematics and Physics, Shanghai University of Electric Power, Shanghai, 201203 (P. R. China).

**Table of content**

[Section S1 Reaction mechanism 3](#_Section_S1_Reaction)

[Section S2 Methods 4](#_Section_S2_Methods)

[Section S3 Result 5](#_Section_S3_Result)

[Section S4 Visualization Tools 7](#_Section_S4_Visualization)

[Section S5 Discussion 9](#_Section_S5_Discussion)

[Section S6 Author Contributions 10](#_Section_S6_Author)

[Section S7 Conflicts of interest 10](#_Section_S7_Conflicts)

[Section S8 Data and code availability 10](#_Section_S8_Data)

[Section S9 References 11](#_Section_S9_References)

# Section S1 Reaction mechanism

The reaction mechanism of the Hiyama coupling reaction is exemplified. The catalytic cycle of the reaction goes through several steps of oxidative addition, transmetalation, cis-trans isomerization, and reductive elimination. The key is to activate the lower Si-R bond for metal-metal exchange. By adding an organic fluorine reagent, such as TBAF, through the interaction of F and Si, +5-valent silicon is formed, thereby activating the C-Si bond.^1^ The detailed mechanism is shown in Fig. S1.

Fig. S1 General mechanism of the Heck reaction.

# Section S2 Methods

**Transformer model**

The transformer model was first introduced in 2017 in an article entitled, “Attention is All You Need”.^2^ The model was originally designed and applied to NMT tasks. When the reaction prediction task is recognized as a language translation task, the transformer model can be applied. The input molecule can be represented as a text sequence, SMILES. Thus, chemical reaction prediction can be considered as a language translation task, that is, translating reactants/products into products/reactants.

In this study, we used the transformer model proposed by Zhang et al. as a baseline model.^3^ The transformer model allows the parallel processing of entire sequences, thus significantly increasing the speed of sequence deep learning models. Then, it introduces an “attention mechanism” that can track relationships between strings in very long sequences of text in both the forward and reverse directions. During training, the encoder module of the transformer receives and processes the complete input string. However, the decoder receives a masked version of the output string (one token at a time) and tries to establish a mapping between the encoded attention vector and the expected result. The encoder attempts to predict the next token and corrects differences between its output and the expected result. This feedback allows the converter to modify the parameters of the encoder and decoder and gradually create the correct mapping between the input and output texts.

Moreover, the more training data and parameters the converter has, the more likely it is to maintain coherence and consistency over longer text sequences. Therefore, data enhancement strategies are often used in such data-driven models.

# Section S3 Result

**Ten-fold cross-validation data for five coupling reactions.** In this work, we derived five coupled reaction datasets from Buchwald-Hartwig, Chan-Lam, Kumada, Hiyama, and Suzuki based on name and structure searches in the "Reaxys" database.^4^ To avoid chance, we used a 10-fold cross-validation method to divide the data. The following five tables are the accuracy rates of the five types of reactions after dividing the data by the 10-fold cross-validation method.

Table.S1 Ten-fold cross-validation accuracy for Hiyama reactions.

| Hiyama Dataset | Accuracy (%) | | | | | | | | | |  |
| --- | --- | --- | --- | --- | --- | --- | --- | --- | --- | --- | --- |
|  | K1 | K2 | K3 | K4 | K5 | K6 | K7 | K8 | K9 | K10 | average |
| raw data | 23.67 | 21.74 | 23.67 | 25.60 | 24.15 | 29.47 | 27.54 | 26.70 | 20.39 | 26.70 | 24.96 |
| Augmented halogen | 44.44 | 37.68 | 34.78 | 43.48 | 43.96 | 45.89 | 39.13 | 34.47 | 33.98 | 36.89 | 39.47 |
| Augmented silicon | 48.31 | 35.75 | 35.75 | 47.34 | 45.41 | 46.38 | 39.61 | 42.72 | 41.26 | 39.32 | 42.19 |
| Augmented halogen  + silicon | 49.47 | 45.89 | 39.13 | 52.17 | 48.31 | 53.14 | 44.44 | 45.63 | 42.71 | 44.66 | 46..56 |

| Buchwald-Hartwig  Dataset | Accuracy (%) | | | | | | | | | |  |
| --- | --- | --- | --- | --- | --- | --- | --- | --- | --- | --- | --- |
|  | K1 | K2 | K3 | K4 | K5 | K6 | K7 | K8 | K9 | K10 | average |
| raw data | 41.63 | 32.13 | 31.90 | 38.46 | 44.80 | 27.38 | 33.94 | 43.89 | 36.65 | 30.16 | 30.09 |
| Augmented halogen | 49.32 | 43.67 | 44.57 | 46.15 | 52.94 | 47.74 | 45.25 | 50.45 | 44.80 | 43.31 | 46.82 |

Table. S2 Ten-fold cross-validation accuracy for Buchwald-Hartwig reactions.

Table. S3 Ten-fold cross-validation accuracy for Cham-Lam reactions.

| Cham-Lam Dataset | Accuracy (%) | | | | | | | | | |  |
| --- | --- | --- | --- | --- | --- | --- | --- | --- | --- | --- | --- |
|  | K1 | K2 | K3 | K4 | K5 | K6 | K7 | K8 | K9 | K10 | average |
| raw data | 64.71 | 57.58 | 63.26 | 61.55 | 65.34 | 61.93 | 66.48 | 55.03 | 56.93 | 57.12 | 60.99 |
| Augmented halogen | 68.50 | 63.45 | 65.72 | 68.37 | 62.88 | 70.64 | 66.86 | 66.60 | 61.48 | 66.23 | 66.07 |

Table. S4 Ten-fold cross-validation accuracy for Kumada reactions.

| Kumada Dataset | Accuracy (%) | | | | | | | | | |  |
| --- | --- | --- | --- | --- | --- | --- | --- | --- | --- | --- | --- |
|  | K1 | K2 | K3 | K4 | K5 | K6 | K7 | K8 | K9 | K10 | average |
| raw data | 78.99 | 79.81 | 77.74 | 79.81 | 81.16 | 82.09 | 78.88 | 72.75 | 78.76 | 75.23 | 78.52 |
| Augmented boron | 80.85 | 80.43 | 81.26 | 80.85 | 81.88 | 82.82 | 80.95 | 79.90 | 79.90 | 78.76 | 80.76 |
| Augmented Grignard  reagent | 84.68 | 82.30 | 83.02 | 78.78 | 84.37 | 83.33 | 83.44 | 82.80 | 81.87 | 81.35 | 82.59 |
| Augmented halogen  + Grignard reagent | 85.40 | 82.61 | 83.33 | 85.30 | 84.78 | 84.68 | 84.37 | 83.01 | 81.45 | 81.66 | 83.66 |

Table. S5 Ten-fold cross-validation accuracy for Suzuki reactions.

| Suzuki Dataset | Accuracy (%) | | | | | | | | | |  |
| --- | --- | --- | --- | --- | --- | --- | --- | --- | --- | --- | --- |
|  | K1 | K2 | K3 | K4 | K5 | K6 | K7 | K8 | K9 | K10 | average |
| raw data | 95.05 | 94.42 | 95.61 | 93.47 | 93.99 | 94.44 | 94.38 | 92.81 | 94.24 | 94.90 | 94.33 |
| Augmented boron | 95.26 | 95.43 | 96.05 | 95.92 | 95.15 | 95.87 | 95.29 | 95.15 | 95.53 | 95.35 | 95.50 |
| Augmented halogen | 96.82 | 95.73 | 97.19 | 96.03 | 96.04 | 96.47 | 95.62 | 95.98 | 96.27 | 95.92 | 96.21 |
| Augmented halogen  + boron | 97.79 | 96.26 | 97.84 | 96.94 | 97.45 | 96.93 | 96.43 | 91.20 | 97.24 | 96.72 | 96.48 |

# Section S4 Visualization Tools

In this section, we generated the plots of reactant molecules belonging to raw datasets and augmented datasets using UMAP, which represents molecules as Morgan fingerprints to create a two-dimensional representation of high-dimensional data distributions.^5,6^ In addition, the program for drawing the UMAP is publicly available on GitHub.


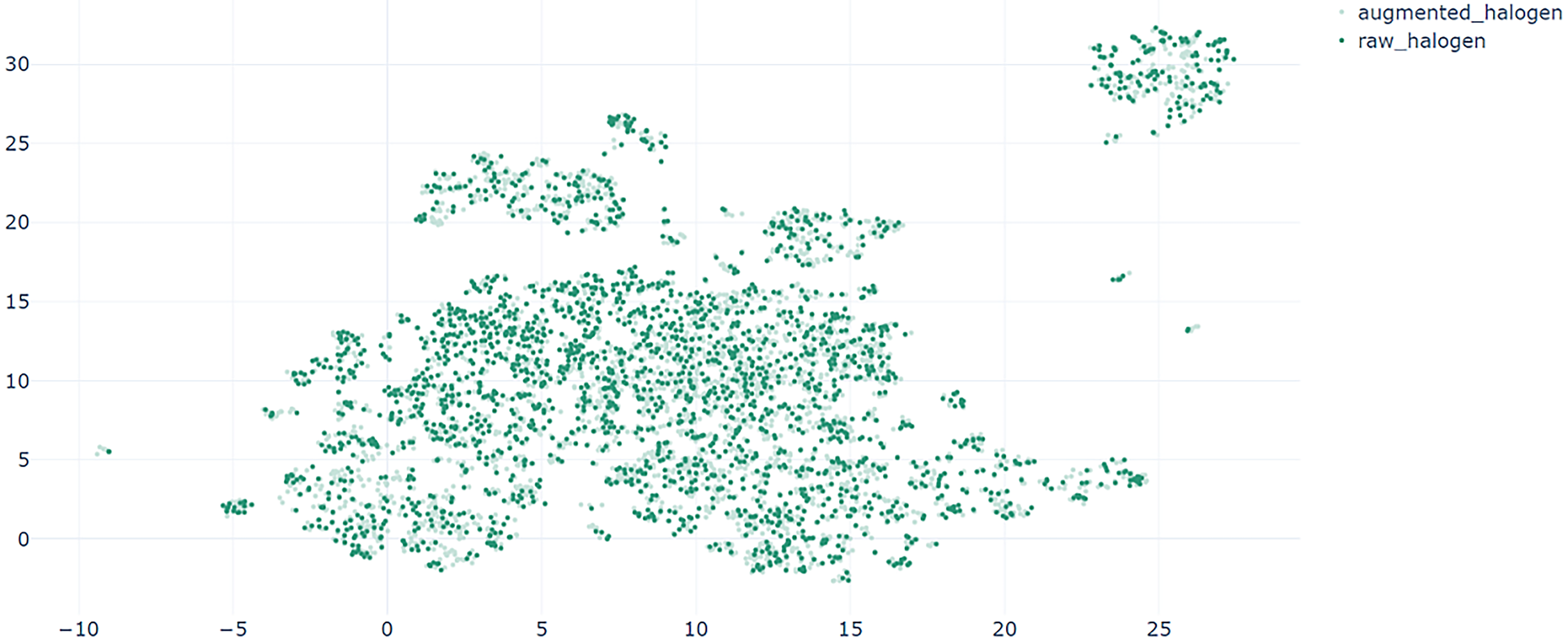
we generated the UMAP plot of Buchwald-Hartwig reactions in Fig. S2, which has only halogen-containing molecules that can be augmented singly. The halogen-containing molecules generated by virtual data augmentation (light green) are still near the raw datasets (green).

Fig. S2 UMAP map of Buchwald-Hartwig coupling reaction before and after virtual data augmentation.

As Fig. S3 demonstrated that the Grignard reagent-containing molecules generated by virtual data augmentation occurring in the training set of Kumada reaction (light pink) are close to the Kumada raw datasets (pink), and the halogen-containing molecules generated by virtual data augmentation occurring in the training set of Kumada reaction (light yellow) are close to the Kumada raw datasets(yellow).


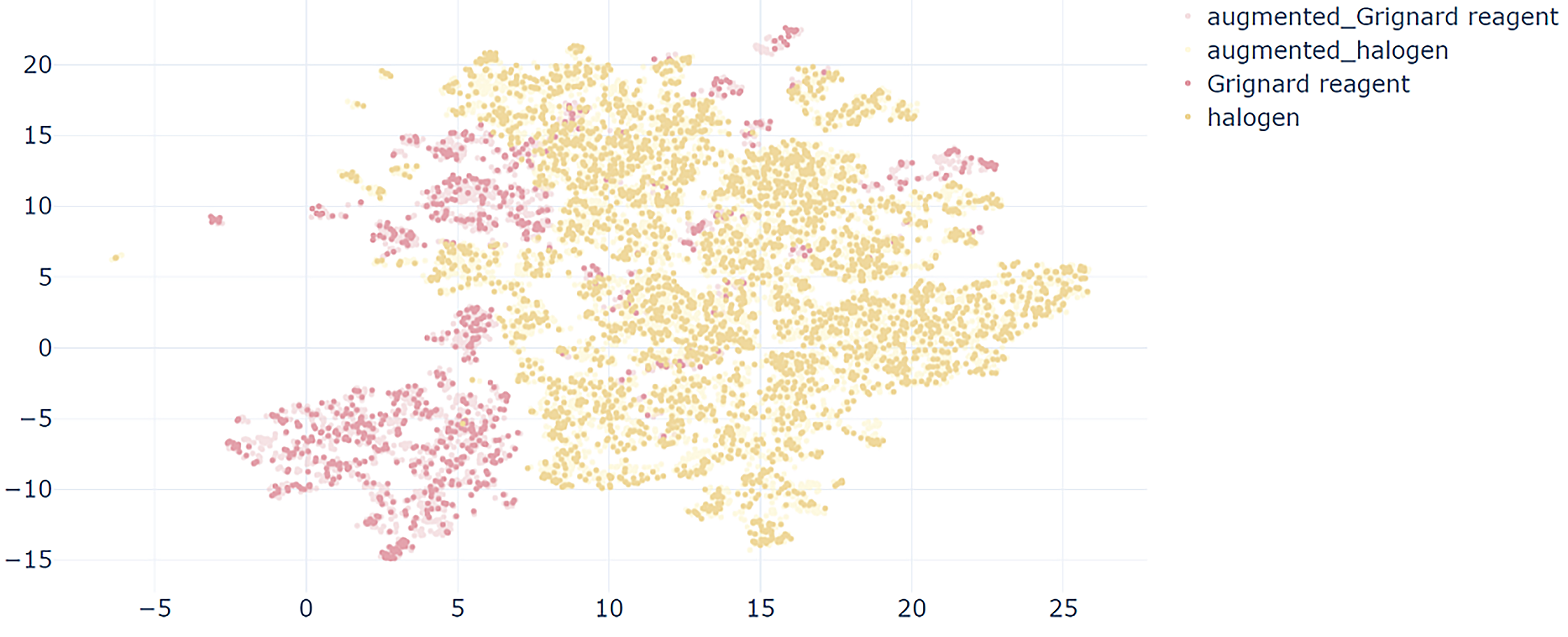


Fig. S3 UMAP map of Kumada coupling reaction before and after virtual data augmentation.

As Fig. S4 demonstrated that the boron-containing molecules generated by virtual data augmentation occurring in the training set of Suzuki reaction (light pink) are close to the Suzuki raw datasets (pink), and the halogen-containing molecules generated by virtual data augmentation occurring in the training set of Suzuki reaction (light purple) are close to the Suzuki raw datasets(purple).


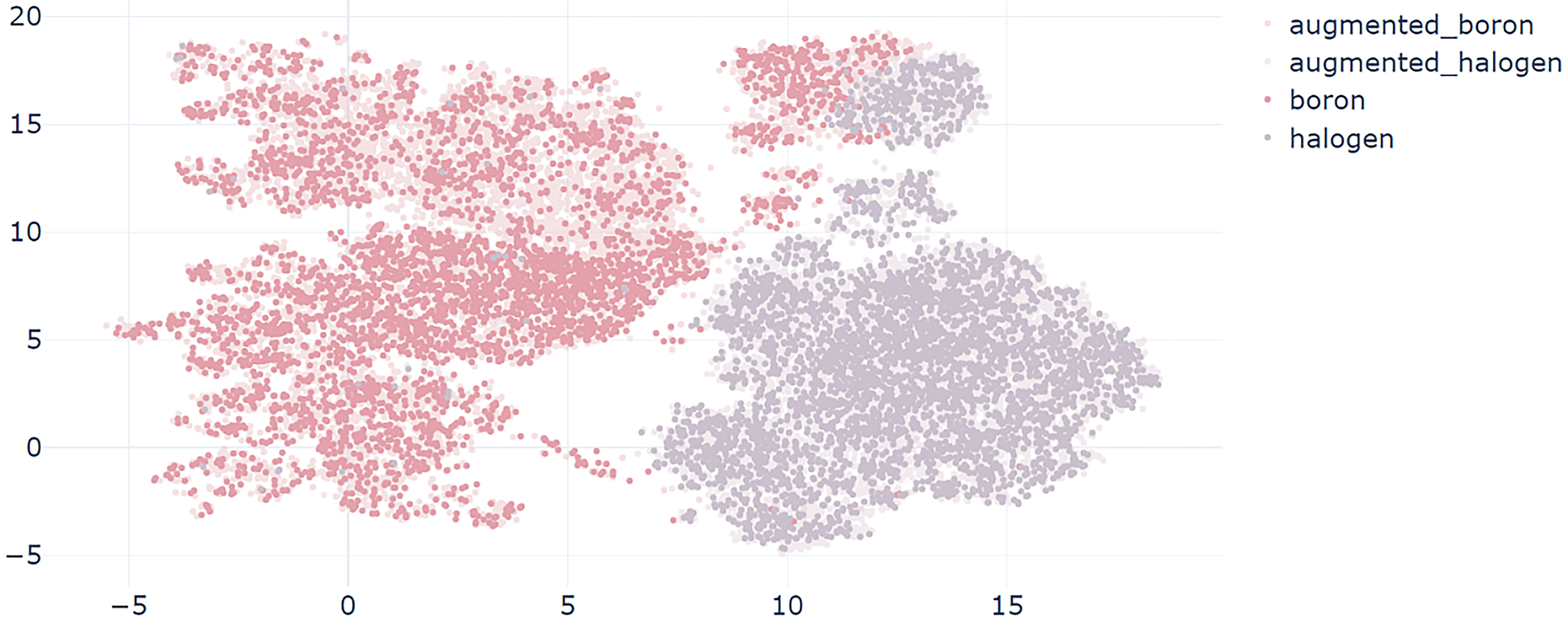


Fig. S4 UMAP map of Suzuki coupling reaction before and after virtual data augmentation.

# Section S5 Discussion

To understand the model training, we conduct several experiments in which the different number of training data sets were randomly selected to monitor their predictive performance in the transformer-baseline model. Different from other tests, this test is based on the Suzuki reaction, which is the largest dataset of all our self-built datasets. We aimed at the one-fold set of Suzuki reactions and randomly sampled 1k, 3k, 5k, 7k, 10k, 30k, and 60k training datasets from raw data. Then these datasets were augmented according to the reactants containing halogen and boron. On the one hand, the transformer model’s performance is affected by the size of the training set. For instance, as shown in Table S6, sampled 1k reactions from the Suzuki dataset, the transformer-baseline model only provided the smallest performance of 1.20% based on the raw dataset, even if integrated with virtual data augmentation, this model reached 1.58%. In contrast, we sampled 10k raw data, and the model calculated top-1 performance of 83.57% and increased by nearly 2.3% after applying the virtual data augmentation method. With the augmentation of the training set, the performance of the model has been significantly improved, demonstrating that direct data augmentation can be great for improving the model’s performance. On the other hand, the effect of virtual data augmentation does not always increase accompanied by the augment of the training set amount. For example, when training on 1k raw data and relevant augmented data, the transformer-baseline model increased by 0.4% before and after applying virtual data augmentation. The gap of the augmented 3k training dataset reaches the largest increment of 15%. While sampled dataset surpasses the 5k dataset, the virtual data augmentation method keeps steady gradually, and there is no obvious upward trend. Therefore, even though the transformer-baseline model is faced with more samples, the transformer-baseline model may be overused and fail to gain new knowledge from chemical reactions.

Table S6. Accuracy of Suzuki reaction datasets of different sizes in the transformer-baseline model before and after data augmentation.

| Suzuki Dataset | Accuracy（%） | | | | | | |
| --- | --- | --- | --- | --- | --- | --- | --- |
|  | 1k | 3k | 5k | 7k | 1w | 3w | 6w |
| raw data | 1.20 | 45.04 | 69.50 | 78.34 | 83.57 | 91.17 | 93.13 |
| Augmented data | 1.58 | 60.38 | 74.52 | 81.11 | 85.87 | 93.84 | 95.90 |

# Section S6 Author Contributions

These authors contributed equally: X. W. and Y. Z. and H. D. designed the research project. X. W., Y. Z., J. Y., Y. W., X. W., Z. W. collected literature and established a self-built dataset. H. Q., C. Z. designed and trained the models. X. W. and Y. Z. analyzed data and wrote the manuscript. All authors discussed the results and approved the manuscript.

# Section S7 Conflicts of interest

The authors declare no competing financial interest.

# Section S8 Data and code availability

The dataset (pretraining and self-built) and the code are available from: <https://github.com/hongliangduan/Virtual-data-augmentation-methood-for-reaction-prediction-in-small-dataset-scenario> Python 3.7 version to write the program. RDKit 2020.09.5 version to process programs.^7^ The map was drawn using the TMAP open-source software.^8^

# Section S9 References

1. Li, J.J. Hiyama cross-coupling reaction. In: Name Reactions. Springer, Cham. <https://doi.org/10.1007/978-3-319-03979-4_134>(2014).

2. Tetko, I. V., Karpov, P., Bruno, E., Kimber, T. B. & Godin, G. Augmentation Is What You Need!. *ICANN 2019*. **11731**, 831–835, <https://doi.org/10.1007/978-3-030-30493-5_79>(2019).

3. Zhang, C. Y., Cai, X., Qiao, H. R., Zhang, Y., Wu, Y. J., Wang, X. Q., Xie, H. Y., Lou, F. & Duan, H. L. Self-supervised molecular pretraining strategy for reaction prediction in low-resource scenarios. Preprint at  [https://doi.org/10.26434/chemrxiv-2021-fxvwg](%20https://doi.org/10.26434/chemrxiv-2021-fxvwg%20)(2021).

4. [http://www.rdkit.org](http://www.rdkit.org/).

5. McInnes, L., Healy, J. & Melville, J. Umap: Uniform manifold approximation and projection for dimension reduction. Preprint at https://doi.org/10.48550/arXiv.1802.03426(2018).

6. Becht, E., McInnes, L. & Healy, J. et al. Dimensionality reduction for visualizing single-cell data using UMAP. Nat Biotechnol 37, 38-44, https://doi.org/10.1038/nbt.4314(2019).

7. <http://www.rdkit.org>.

8. <https://tmap.gdb.tools/>
